# Eosinophilic Esophagitis After an Allegra-D Bolus: A Case Report

**DOI:** 10.7759/cureus.20533

**Published:** 2021-12-20

**Authors:** Justin Chuang, Khushbu Patel, Naveena Luke, Jordan Burlen, Ali Nawras

**Affiliations:** 1 Internal Medicine, The University of Toledo Medical Center, Toledo, USA; 2 Gastroenterology and Hepatology, The University of Toledo Medical Center, Toledo, USA

**Keywords:** esophagitis, eosinophilic, eoe, allegra, eosinophilic esophagitis

## Abstract

Eosinophilic esophagitis (EoE) is an immune-mediated disorder that may be related to exposure to additive chemicals in crops, air pollutants, or supplements found within livestock. Co-occurring allergic or atopic diseases including atopic dermatitis, food allergies, and asthma are also commonly seen in 70% of cases and help guide diagnosis. Diagnosis of EoE requires eosinophilic infiltration greater than 15 eosinophils per high power field (HPF) with endoscopic evidence of abnormal esophageal changes. Here, we discuss a rare presentation of food bolus impaction secondary to EoE after ingestion of a nasal decongestant and antihistamine pill that has previously never been described in the literature.

A 22-year-old male with no significant past medical history presented to the emergency department (ED) with a chief complaint of a sudden onset respiratory distress, regurgitation of clear oral secretions, and globus sensation post ingestion of a fexofenadine-pseudoephedrine tablet. Prior to intake of the capsule, the patient was consuming liquids and solids appropriately. The patient was afebrile, hypertensive at 172/114, and found to have a normal heart rate of 88 bpm and a respiration rate of 18 breaths per minute. An esophagogastroduodenoscopy (EGD) was performed, which revealed a fexofenadine-pseudoephedrine capsule at 23 cm from the incisors along with a superficial ulceration at the corresponding level in the esophagus. The foreign body was successfully removed using raptor forceps. Further visualization demonstrated trachealization of the esophagus and furrowing and severe narrowing (< 10mm), which raised suspicion for EoE. Proximal biopsy indicated 16 intraepithelial eosinophils per HPF within the squamous epithelium, likely compatible with EoE. The patient tolerated the procedure well and was discharged on an eight-week course of proton-pump inhibitors.

EoE is defined as an immune-mediated esophageal disease characterized histologically by eosinophil-predominant inflammation. Our patient was reported to have up to 30 eosinophils per HPF from the proximal esophageal biopsy, which satisfies the requirements for an EoE diagnosis. Based on the current literature review, there have been no other reported cases of symptomatic food bolus impaction secondary to EoE after ingestion of antihistamines.

## Introduction

Eosinophilic esophagitis (EoE) is an immune-mediated disorder commonly occurring in adults aged 35 to 45 years or in children aged 5.4 to 9.6 years [1]. The disease is known to cause gastrointestinal symptoms such as dysphagia, reflux, nausea, vomiting, abdominal pain, or food impaction as a result of eosinophilic infiltration of the esophagus [2,3]. The prevalence of food impaction secondary to EoE has significantly risen over time especially in the male Caucasian population [1,2].

EoE occurs secondarily to a presumed combination of genetic and environmental factors [2]. Cytokine-mediated susceptibility or dysfunctional esophageal epithelium could be genetically predisposing factors that impact a person’s vulnerability in developing EoE [2]. Predisposition to EoE may also be related to exposure to additive chemicals in crops, air pollutants, or supplements found within livestock [2]. Co-occurring allergic or atopic diseases including atopic dermatitis, food allergies, and asthma are also commonly seen in 70% of cases and help guide diagnosis [3,4]. Diagnosis of EoE requires eosinophilic infiltration greater than 15 eosinophils per high power field (HPF) with endoscopic evidence of abnormal esophageal changes [1,2]. Generally, food allergens are the widely known etiologies of EoE; hence, treatment plans often consist of a six-food elimination diet along with topical steroids or proton-pump inhibitors to manage and decrease future recurrence of the disease [1,2].

Here, we discuss a rare presentation of food bolus impaction secondary to EoE after ingestion of a nasal decongestant and antihistamine pill that has previously never been described in the literature.

The abstract of this article was previously published in The American Journal of Gastroenterology, Volume 116, in October 2021.

## Case presentation

A 22-year-old male with no significant past medical history presented to the emergency department (ED) with a chief complaint of a sudden-onset respiratory distress, regurgitation of clear oral secretions, and globus sensation post ingestion of a fexofenadine-pseudoephedrine tablet. Prior to intake of the capsule, the patient was consuming liquids and solids appropriately. The patient was afebrile, hypertensive at 172/114, and found to have a normal heart rate of 88 bpm and respiration rate of 18 breaths per minute. He denied any food allergies or previous episodes of bolus impaction. He was not taking any other daily medications nor did he have any history of tobacco or alcohol use. Physical examination was unremarkable. Chest radiograph showed no prevertebral soft tissue or epiglottis swelling. No radiopaque foreign object was visualized. In the ED, the patient was given supportive measures due to concerns of an allergic reaction.

**Figure 1:**
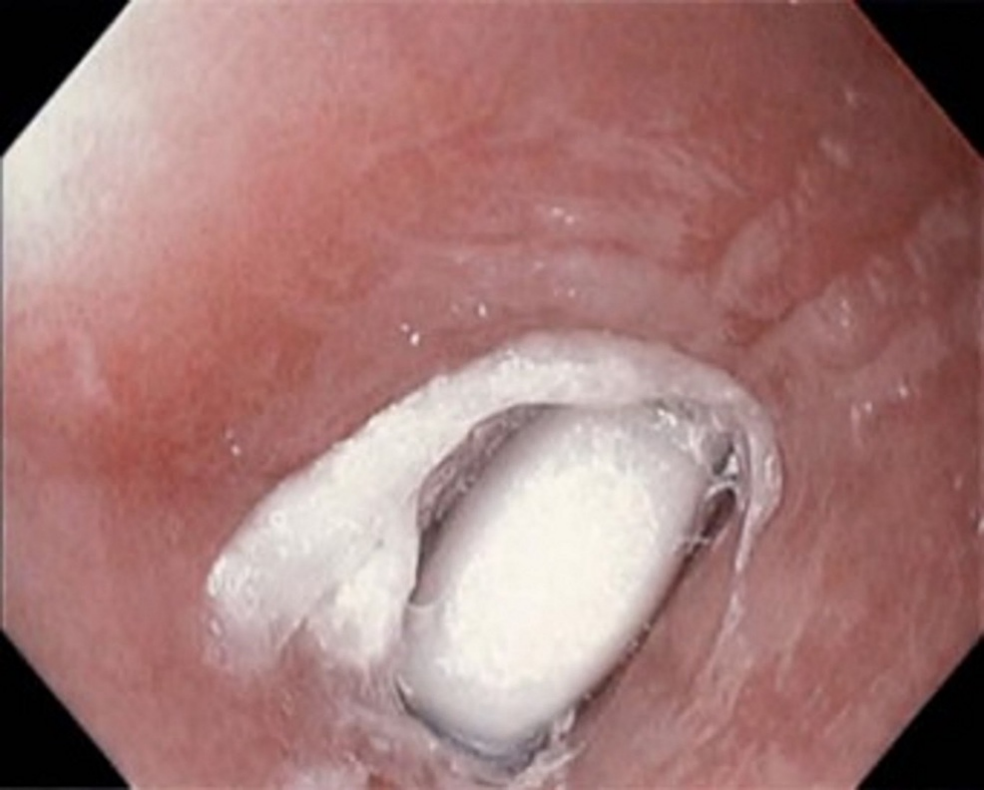
Esophagogastroduodenoscopy revealing a fexofenadine-pseudoephedrine capsule at 23 cm from incisors.

**Figure 2:**
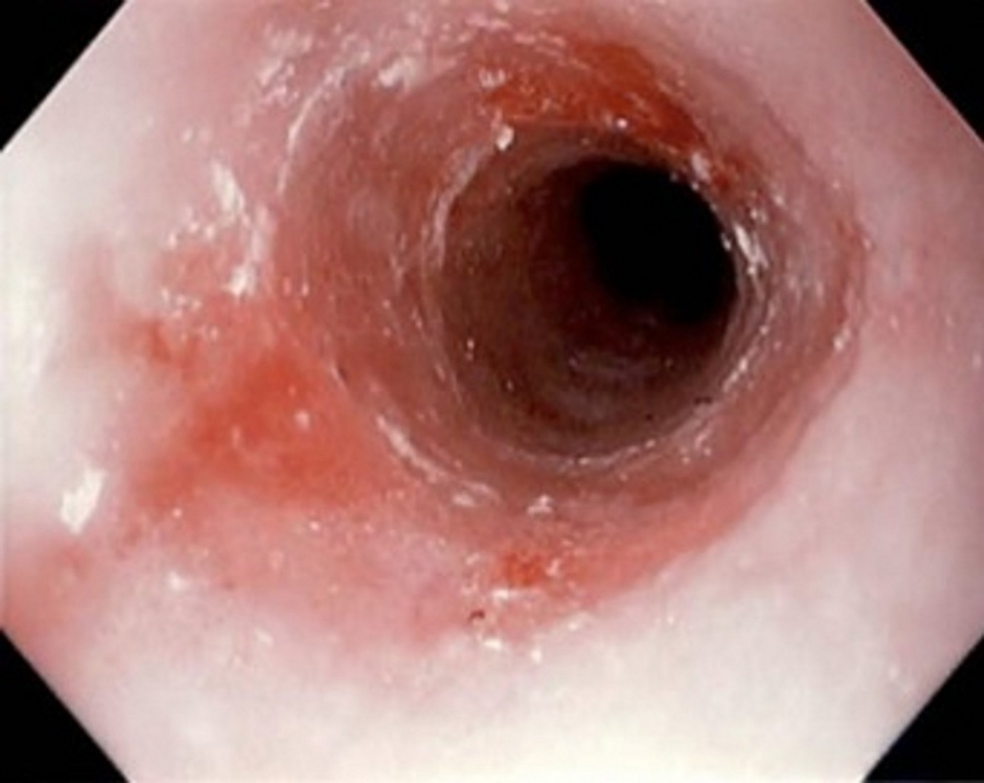
Successful removal of the fexofenadine-pseudoephedrine capsule.

**Figure 3:**
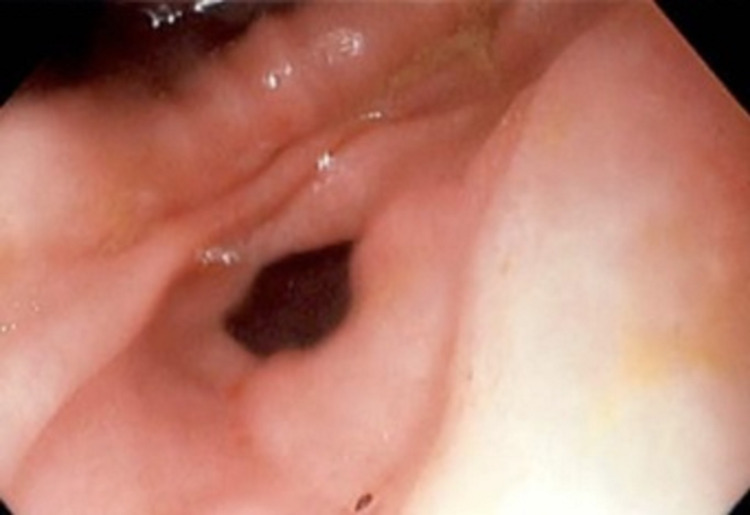
Endoscopic presentation of esophageal narrowing and trachealization

An esophagogastroduodenoscopy (EGD) was performed, which revealed a fexofenadine-pseudoephedrine capsule at 23 cm from the incisors along with a superficial ulceration at the corresponding level in the esophagus (Figure 1). The foreign body was successfully removed using raptor forceps (Figure 2). Further visualization demonstrated trachealization of the esophagus and furrowing and severe narrowing (<10mm), which raised suspicion for EoE (Figure 3). Biopsies were performed at the proximal and distal esophagus to further classify the diagnosis. Proximal biopsy indicated 16 intraepithelial eosinophils per HPF within the squamous epithelium likely compatible with EoE. The patient tolerated the procedure well, and the decision was made to start the patient on an eight-week course of proton-pump inhibitors.

## Discussion

EoE is defined as an immune-mediated esophageal disease characterized histologically by eosinophil-predominant inflammation [2,4]. Eosinophils have varied distribution throughout the gastrointestinal tract. Typically, the highest numbers are found in the cecum and appendix, whereas the squamous epithelium of the esophagus is normally devoid of eosinophils [5]. The accepted qualification for EoE diagnosis is currently an intraepithelial eosinophil count of 15 or more eosinophils per HPF from esophageal biopsies [1,2]. Our patient was reported to have up to 30 eosinophils per HPF from the proximal esophageal biopsy, which satisfies the requirements for an EoE diagnosis.

Currently, it is thought that allergens activate the eosinophils within the esophageal epithelium and trigger the inflammatory cascade and chronic inflammation leads to the classic finding of trachealization of the esophagus [4]. Reported individual or family histories of allergic diseases, including food allergies, eczema, asthma, allergic rhinitis, have been noted in approximately 70% of individuals with EoE. However, adults presenting with new-onset EoE are less likely to present with an allergic history or peripheral eosinophilia [6]. Only a minority of EoE patients have presented with food anaphylaxis [4]. Similarly, our patient had no history of atopic disease or any previous history of anaphylactic episodes.

This patient’s endoscopic imaging was also consistent with typical EoE findings including trachealization of the esophagus and furrowing. EoE can present with difficulty swallowing, decreased mobility through the esophagus, or as an acute bolus obstruction that can necessitate emergency medical attention [7]. Our patient presented similarly to the ED with subsequent respiratory distress and regurgitation.

EoE is understood to be Th2 antigen-driven, with food allergens or environmental pollutants as the primary source of the derived antigens [4,7]. Our patient, however, experienced symptoms following ingestion of a fexofenadine-pseudoephedrine capsule. Allegra-D (fexofenadine hydrochloride combined with pseudoephedrine hydrochloride) belongs to the antihistamine/decongestant drug class. Fexofenadine is a selective H1-receptor antagonist that has even been shown to induce eosinophil apoptosis [8].

To our knowledge, there are no other reported cases of symptomatic food bolus impaction secondary to EoE after ingestion of antihistamines. A thorough search was conducted on PubMed, Cochrane Library, and Google Scholar. Keywords included “eosinophilic esophagitis, food impaction, Allegra-D, fexofenadine and eosinophilic esophagitis, antihistamines with eosinophilic esophagitis, antihistamine hypersensitivity” where we did not find similar cases. Hence, we are supporting that this case was indeed a rare and interesting case and hope it may provide useful information in the future for gastroenterologists who may encounter a similar patient.

Potential limitations to this case report include the limited evaluation of biopsy specimens due to fragile specimens that might have not survived processing. The pathologist noted that especially for the distal segment, it was difficult to determine if the scant number of eosinophils present in the biopsy was representative of the entire distal esophagus.

## Conclusions

Practitioners should maintain a high index of suspicion for EoE in adult male patients who present with dysphagia or food impactation that is unresponsive to antacids, even with little to no history of atopy. It is critical to rule out other differentials such as achalasia, stricture, esophageal webs, and cancer with appropriate diagnostic tests and ultimately rule in EoE with esophageal biopsy. Typical first-line treatments include allergen avoidance after elimination diet trial, proton-pump inhibitors, and topical steroids. Combination therapy may be ultimately required to reduce inflammation and control symptoms.
